# Bioinformatics Analysis of Molecular Interactions between Endoplasmic Reticulum Stress and Ferroptosis under Stress Exposure

**DOI:** 10.1155/2023/9979291

**Published:** 2023-03-30

**Authors:** Weihao Zhu, Yingmin Li, Meili Li, Jingmin Liu, Guowei Zhang, Xiaoying Ma, Weibo Shi, Bin Cong

**Affiliations:** Hebei Key Laboratory of Forensic Medicine, Collaborative Innovation Center of Forensic Medical Molecular Identification, College of Forensic Medicine, Hebei Medical University, No. 361 Zhongshan Dong Road, 050017 Shijiazhuang, China

## Abstract

Stress has become a universal biological phenomenon in the body, which leads to pathophysiological changes. However, the molecular network interactions between endoplasmic reticulum (ER) stress and ferroptosis under stressful conditions are not clear. For this purpose, we screened the gene expression profile of GSE173795 for intersection with ferroptosis genes and screened 68 differentially expressed genes (DEGs) (63 up-regulated, 5 down-regulated), mainly related to lipid and atherosclerosis, autophagy—animal, mitophagy—animal, focal adhesion, DNA replication, proteasome, oocyte meiosis, toll-like receptor signaling pathway, cell cycle, etc. Immune infiltration analysis revealed that stress resulted in decreased B cells memory, T cells CD8 and T cells CD4 memory resting, monocytes, macrophages M2, and increased B cells naive, T cells follicular helper, and macrophages M1. 19 core-DEGs (ASNS, TRIB3, ATF4, EIF2S1, CEBPG, RELA, HSPA5, DDIT3, STAT3, MAP3K5, HIF1A, HNF4A, MAPK14, HMOX1, CDKN1A, KRAS, SP1, SIRT1, EGFR) were screened, all of which were up-regulated DEGs. These biological processes and pathways were mainly involved in responding to ER stress, lipid and atherosclerosis, cellular response to stress, cellular response to chemical stress, and regulation of DNA-templated transcription in response to stress, etc. Spearman analysis did not find MAPK14 to be significantly associated with immune cells. Other core-DEGs were associated with immune cells, including B cells naive, T cells follicular helper, and monocytes. Based on core-DEGs, 283 miRNAs were predicted. Among the 22 miRNAs with highly cross-linked DEGs, 11 had upstream lncRNA, mainly targeting STAT3, SP1, CDKN1A, and SIRT1, and a total of 39 lncRNA were obtained. 85 potential drugs targeting 11 core-DEGs were identified and were expected to be potential immunotherapeutic agents for stress injury. Our experiments also confirmed that Liproxstatin-1 alleviates common cross-linked proteins between ER stress and ferroptosis. In conclusion, our study explored the molecular mechanisms and network interactions among stress—ER stress—ferroptosis from a novel perspective, which provides new research ideas for studying stressful injury.

## 1. Introduction

Stress is a systemic and non-specific adaptive response of the body to the stimuli from various stressors in the internal and external environment [1]. When the stressor is too intense and persistent, although the various responses of the organism still have some adaptive defensive significance [2], the main mechanisms of stress are those that lead to an increase in homeostatic load, hypoglycemia, ischemia, and hypoxia, imbalance of Ca^2+^ levels in the body or disturbance of regulatory factors and hormone levels. These deregulate the redox balance of the endoplasmic reticulum (ER), leading to the accumulation of unfolded or misfolded proteins in the ER, triggering ER stress, and activating the body's unfolded protein reaction (UPR) [3–5]. UPR can transmit unfolded protein signals through the ER membrane by activating three ER transmembrane proteins in vivo: the type I transmembrane protein kinase R-like ER kinase (PERK), inositol requiring enzyme 1 (IRE1), and active transcription factor 6 (ATF6), which mediate distinct signaling pathways at the transcriptional and translational levels [5, 6]. And relieve ER stress through three functions: (1) C/EBP homologous protein (CHOP)/growth arrest and DNA damage-inducible gene 153 (GADD153) activation pathway: when misfolded or unfolded proteins in the ER cannot be corrected, ATF6 continues to be expressed, activating the expression of the downstream apoptotic gene CHOP/GADD15. CHOP can inhibit the expression of the molecular chaperone glucose-regulated protein 78 (GRP78)/immunoglobulin heavy chain-binding protein (BiP) and the anti-apoptotic gene B-cell lymphoma-2 (BCL-2), deplete glutathione (GSH) increase the ER-derived reactive oxygen species (ROS), and cause apoptosis [3, 7]. (2) C-Jun N-terminal kinase (JNK) pathway: prolonged activation of the core receptor pathway of IRE1 will activate the TRAF-ASK1-JNK pathway and induce apoptosis [3, 8]. (3) Caspase-12 pathway: caspase-12 is a specific ER stress-related protease, and activated caspase-12 undergoes a cascade reaction that ultimately leads to apoptosis [7–9].

Ferroptosis is a newly discovered iron-dependent, non-apoptotic, programmed cell death mode [10], caused mainly by iron deposition, of glutathione peroxidase (GPX4) inactivation, and polyunsaturated fatty acids accumulation, accompanied by massive lipid peroxides production [11]. It differs morphologically, biochemically, and genetically from cell death mechanisms such as autophagy, apoptosis, necrosis, programmed necrosis, and pyroptosis [10, 11]. Its pathways mainly involve the following: system Xc-/GSH/GPX4 (GPX4-GSH-Cysteine) regulation, mevalonate pathway, ferroptosis suppressor protein 1/coenzyme Q10/NADPH pathway (FSP1-CoQ10-NADPH), and nuclear factor erythroid-2-related factor 2 (Nrf2)/ARE-GPX4 pathway [12]. In recent years, evidence of iron deposition or abnormal iron homeostasis has been found in stress injury [13, 14], and researches on the mechanism of ferroptosis in stress have been gradually developed [15, 16]. However, there are few studies on the relationship between ER stress and ferroptosis in the models of stress-induced body injury, and their molecular interactions are still unclear. With the development of high-throughput sequencing and microarray technology, it has become a trend to use bioinformatics and gene chip technology to study the occurrence and development of various injuries and diseases [17, 18]. With the characteristics of comprehensive data and large sample size, gene chips provide the possibility to study the molecular combination of ER stress and ferroptosis.

In summary, we attempted to explore the mechanisms and molecular network interactions of stress—ER stress—ferroptosis by using bioinformatics technology to further elucidate the occurrence and development of stress injury. Therefore, in this study, we screened the gene expression profiles of ER stress in the national center for biotechnology information-gene expression omnibus (NCBI-GEO) database (https://www.ncbi.nlm.nih.gov/geo/), took differentially expressed genes (DEGs) that intersected with ferroptosis genes, and used bioinformatics to analyze the molecular mechanisms of DEGs in the occurrence and development of ER stress and ferroptosis to provide some new ideas for studying stress injury.

## 2. Materials and Methods

### 2.1. Data Source

The gene expression profile of GSE29929 was obtained from the NCBI-GEO database and sequenced by GPL1261 platform [Mouse430_2] Affymetrix Mouse Genome 430 2.0 Array. It was used to investigate the gene expression difference during ER stress (tunicamycin (TM) induced) in mice. Three control samples and four stressed samples of wild type (WT) mice were selected, totaling seven. We also collected 288 ferroptosis genes from the FerrDb database (http://zhounan.org/ferrdb/legacy/index.html), including 108 driver genes, 69 suppressor genes, and 111 marker genes (which is larger than the gene count (259), because of 28 multi-annotated genes). NCBI-GEO and FerrDb belong to international public databases, which are used to help researchers query and download genetic data.

### 2.2. Screening DEGs

GEO2R analysis tool was employed to compare the gene expression profiles of stressed samples and controls samples to obtain DEGs. The DEGs meeting |logFC| > 1 and *p*-value <0.05 were screened, in which the DEGs with logFC > 1 were regarded as up-regulated genes, and the DEGs with logFC < −1 were regarded as down-regulated genes. The common DEGs of GSE29929 and ferroptosis genes were screened by the Venn tool (http://bioinformatics.psb.ugent.be/webtools/Venn/), and the heat map of the common DEGs expression was drawn with HemI 2.0.

### 2.3. Function and Pathway Enrichment Analysis

We use the database for annotation, visualization and integrated discovery (DAVID) database (https://david.ncifcrf.gov/home.jsp) to systematically annotate the biological functions of DEGs. Select gene ontology (GO) function analysis and Kyoto encyclopedia of genes and genomes (KEGG) pathway enrichment analysis, with *p*-value <0.01 and FDR < 0.01 statistically significant. The analysis items include biological process (BP), molecular function (MF), cell component (CC), and signal pathway. Subsequently, we also used gene set enrichment analysis (GSEA) to conduct pathway enrichment analysis on DEGs, screen enrichment pathways with *p*-value and adjust *p*-value less than 0.01, and select the top six.

### 2.4. Immune Infiltration Analysis

To confirm the correlation between ER stress and immune cells, the expression data of GSE29929 were uploaded to the CIBERSORTx (https://cibersortx.stanford.edu/) for immune infiltration analysis. The analysis conditions were set as follows: signature matrix file: LM22; permutations for significance analysis: 1000; check “enable batch correction”. According to the screening results of *p*-value <0.05, the immune cell types that did not participate were eliminated. Draw the composition diagram of immune cells in each sample, the differential expression diagram of immune cells in the control group and the stress group, and the correlation diagram of immune cells. Meanwhile, principal component analysis (PCA) analysis was performed to verify whether the stress group could be distinguished from the control group based on the difference in immune cell infiltration.

### 2.5. Core-DEGs Screening, Function, and Pathway Enrichment Analysis

The protein–protein interaction (PPI) network diagram was constructed by the STRING tool (https://string-db.org/), and then the core-DEGs were screened using the molecular complex detection (MCODE) function of Cytoscape 3.9.1 software. The setting conditions of the MCODE application were: degree cutoff = 2, node score cutoff = 0.2, *k*-core = 2, max.depth = 100. Upload the screened core-DEGs to the DAVID database for GO function and KEGG pathway analysis. Also, Metascape (http://metascape.org/gp/index.html#/main/step1) was employed to re-identify the BP and pathway enrichment of core-DEGs, and the top 20 were selected on the premise of *p*-value <0.01. Finally, PCA analysis verified whether the stress and control groups could be identified based on the expression difference of core-DEGs.

### 2.6. Core-DEGs and Immune Cells

To understand the role of core-DEGs in immune infiltration, we conducted a spearman correlation analysis between core-DEGs and immune cells based on the results of immune infiltration analysis to determine whether 19 core-DEGs are related to immune infiltration. It is statistically significant to select *p*-value <0.1.

### 2.7. Core-DEGs–miRNA–lncRNA Interactive Network

We use miRWalk 3.0 (http://mirwalk.umm.uni-heidelberg.de/) to predict that core-DEGs target key miRNAs, and cross the prediction results of MiRTarBase and miRWalk databases to ensure the accuracy of the results. The screening results were based on *p*-value <0.05, the seed sequence length was greater than 7, and the target gene binding region was 3′UTR. The CC, MF, BP, and biological pathway of miRNA were enriched and analyzed by Funrich software. Subsequently, StarBase v2.0 (https://starbase.sysu.edu.cn/) was used to identify the upstream molecules lncRNAs of miRNA, and the highest reliability was selected as the standard to analyze the core-DEGs–miRNA–lncRNA interaction network.

### 2.8. Potential Therapeutic Agents for Stress

Based on the core-DEGs, we use the DGIdb database (https://dgidb.org/) of gene–drug interactions to identify potential therapeutic drugs (preset filters check “approved” and “immunotherapies”) and display the interactions between drugs, genes, and immune cells.

### 2.9. Verify ATF4, EIF2S1, HSPA5, and MAP3K5

We randomly selected four core-DEGs (ATF4, EIF2S1, HSPA5, and MAP3K5) from 19 core-DEGs for validation in the mouse model.

Animal model construction: 20 C57 mice (male, 20 ± 2 g) at 6–8 weeks were provided by Beijing Weitong Lihua Experimental Animal Technology Co., Ltd. with the certificate number SCXK (Beijing) 2021-0006. Adaptive feeding for 7 days, free diet and drinking water, 12 hours light–dark cycle rhythm, temperature 20°C–25°C, humidity 50 ± 5%. This study was approved by the Laboratory Animal Management Committee of Hebei Medical University, making every effort to reduce the number of animals and minimize pain and suffering. They were randomly divided into four groups: control group, TM group, TM + Liproxstatin-1 (Lip-1, specific inhibitors of ferroptosis) group, Lip-1 group, with five mice in each group. TM of 1 mg/kg was injected intraperitoneally in the TM group; in the TM + Lip-1 group, 10 mg/kg Lip-1 was injected again 1 hour after intraperitoneal injection of 1 mg/kg TM; Lip-1 group received intraperitoneal injection of 10 mg/kg Lip-1; the normal group was given the same amount of normal saline. All groups were put to death after neck dislocation 24 hours after injection, and the left lobe of liver tissue was extracted and stored in 4% formaldehyde.

Histopathology and immunohistochemistry: fixed liver tissue, dehydrated by ethanol, transparent by xylene, paraffin embedding, 4 *μ*m serial sectioning, routine hematoxylin eosin (HE) staining, neutral resin sealing, and Aperio ScanScope CS2 scanner (Leica, Germany) was used to observe the pathological changes. Immunohistochemical verification of four proteins, namely ATF4, EIF2S1, HSPA5, MAP3K5, and three slices of each protein on each paraffin-embedded block, drying in a 60°C incubator, dewaxing to water, antigen repair, endogenous peroxidase blocking, goat serum blocking, antibody (1 : 100) incubation overnight at 4°C, reaction enhancement solution incubation for 40 minutes at 37°C, enhanced enzyme labeled goat anti-mouse/rabbit immunoglobulin G (IgG) polymer incubation for 40 minutes at 37°C, 3,3′-diaminobenzidine (DAB) coloration, hematoxylin re-staining, and film sealing. Aperio ScanScope CS2 scanner was employed for observation, and color deconvolution V9 software was selected for statistical analysis based on average positive intensity.

## 3. Results

### 3.1. DEGs Screening Results

**Figure 1:**
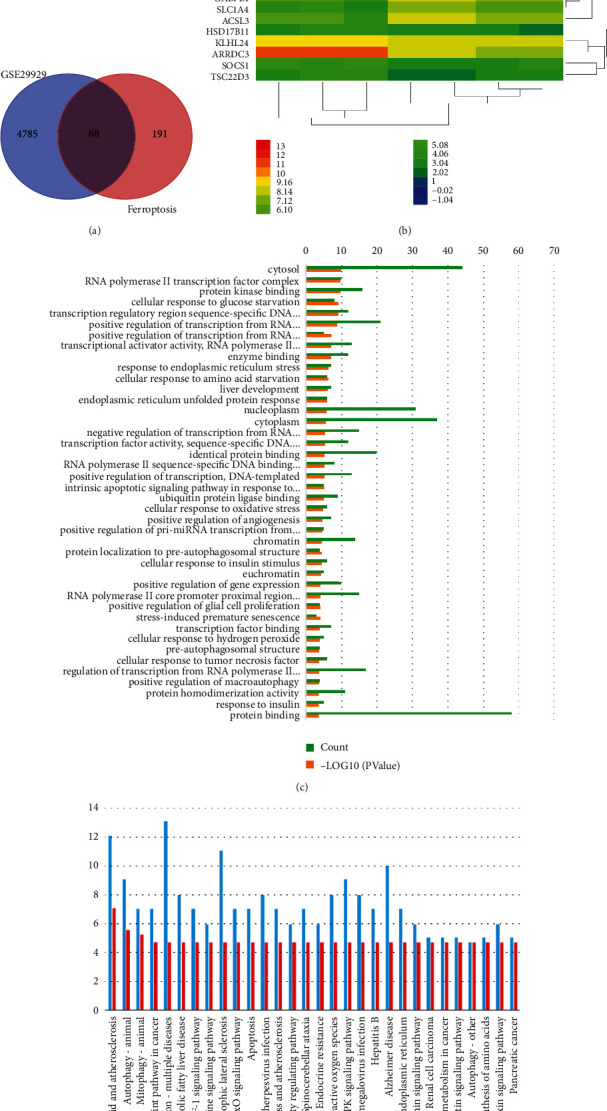
Common DEGs screening, expression changes, and functional and pathway enrichment analysis. (a) Intersection of GSE29929 and ferroptosis; (b) heat map of expression of common DEGs; (c) results of GO functional analysis of common DEGs; (d) results of KEGG pathway enrichment of common DEGs; (e) results of GSEA analysis of common DEGs.

We screened 4853 DEGs (3954 up-regulated and 899 down-regulated) from GSE29929 during ER stress in mice, and 68 common DEGs (63 up-regulated and 5 down-regulated) were screened after crossing with the ferroptosis gene (Figure 1(a), Table 1). It can be seen from the heat map of the expression of 68 common DEGs that there is a significant difference between the control group and the stress group (Figure 1(b)).

### 3.2. Function and Pathway Enrichment Analysis Results

68 DEGs were uploaded to the DAVID, of which 67 were successfully used for GO function analysis and 54 were successfully used for KEGG pathway enrichment analysis. The results of the GO function analysis are shown in Figure 1(c). BP involves 23 aspects, among which the top three of *p*-value are cellular response to glucose starvation, positive regulation of transcription from RNA polymerase II promoter, positive regulation of transcription from RNA polymerase II promoter in response to ER stress. MF has 13 aspects, mainly focusing on protein kinase binding, transcription regulatory region sequence-specific DNA binding, transcriptional activator activity, RNA polymerase II transcription regulatory region sequence-specific binding, etc. CC involves nine aspects, including cytosol, RNA polymerase II transcription factor complex, nucleoplasm, cytoplasm, chromatin, euchromatin, pre-autophagosomal structure, nucleus, and lipid particle. The enrichment results of the KEGG pathway are shown in Figure 1(d), involving 30 pathways, mainly including lipid and atherosclerosis, autophagy—animal, mitophagy—animal, programmed cell death 1 ligand 1 (PD-L1) expression and PD-1 checkpoint pathway in cancer, pathways of neurodegeneration—multiple diseases, etc. The KEGG enrichment analysis conducted by GSEA shows that it mainly involves focal adhesion, DNA replication, proteasome, oocyte meiosis, toll-like receptor signaling pathway, cell cycle (Figure 1(e)).

### 3.3. Immune Infiltration Results

**Figure 2:**
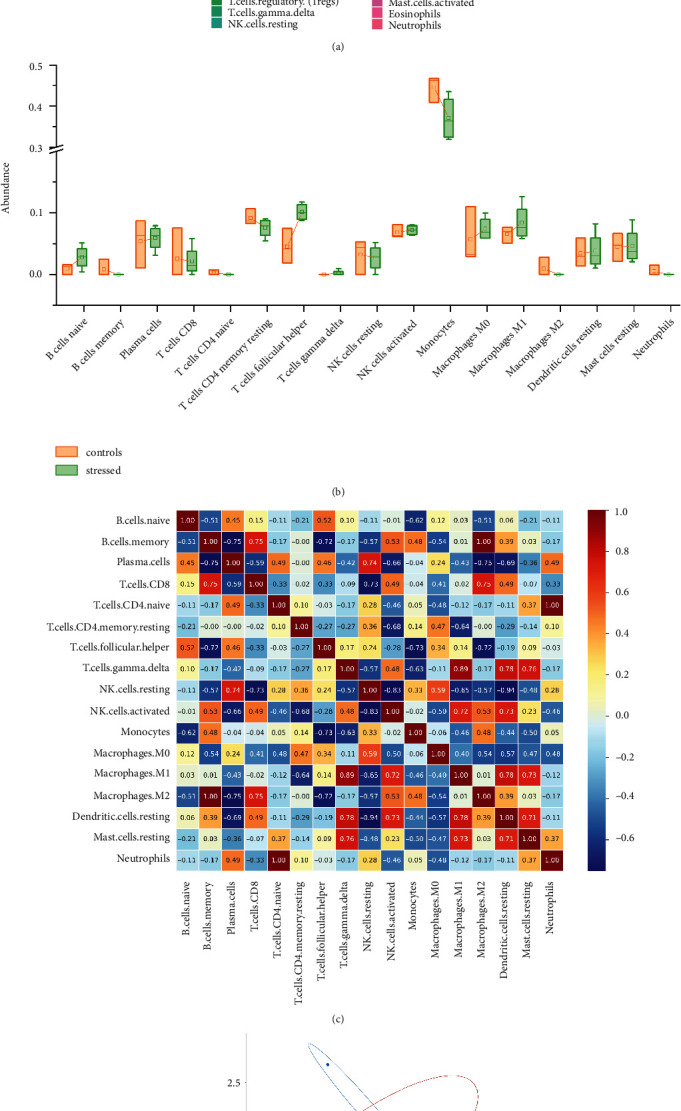
Immune cell infiltration analysis in the control and stress groups. (a) Histogram consisting of 22 immune infiltrating cells of each sample; (b) differences in immune infiltrating cells between the control and stress groups; (c) results of correlation analysis of infiltrating immune cells; (d) results of PCA analysis of immune cell infiltration in the control and stress groups.

Three control samples and four stressed samples were all subjected to immune infiltration analysis. Histograms of the composition of 22 immune cells showed (Figure 2(a)) that monocytes, NK cells activated, macrophages M1, T cells CD4 memory resting, and macrophages M0 were the main infiltrating immune cells. Compared with the control group, B cells memory, T cells CD8 and T cells CD4 memory resting, monocytes, macrophages M2 in the stressed group decreased, while B cells naive, T cells follicular helper, and macrophages M1 increased (Figure 2(b)). The correlation analysis of infiltrating immune cells is shown in Figure 2(c), in which B cells memory and macrophages M2, T cells CD4 naive, and neutrophils have the strongest synergistic effect (*R* = 1). The competition effect of dendritic cells resting and NK cells resting was the significant (*R* = −0.94). Unfortunately, based on the infiltration of immune cells in liver tissue, PCA analysis results showed that *P* = 0.313, *R* = 0.111, which failed to distinguish the control group from the stress group (Figure 2(d)).

### 3.4. Core-DEGs Screening, Function, and Pathway Enrichment Analysis Results

**Figure 3:**
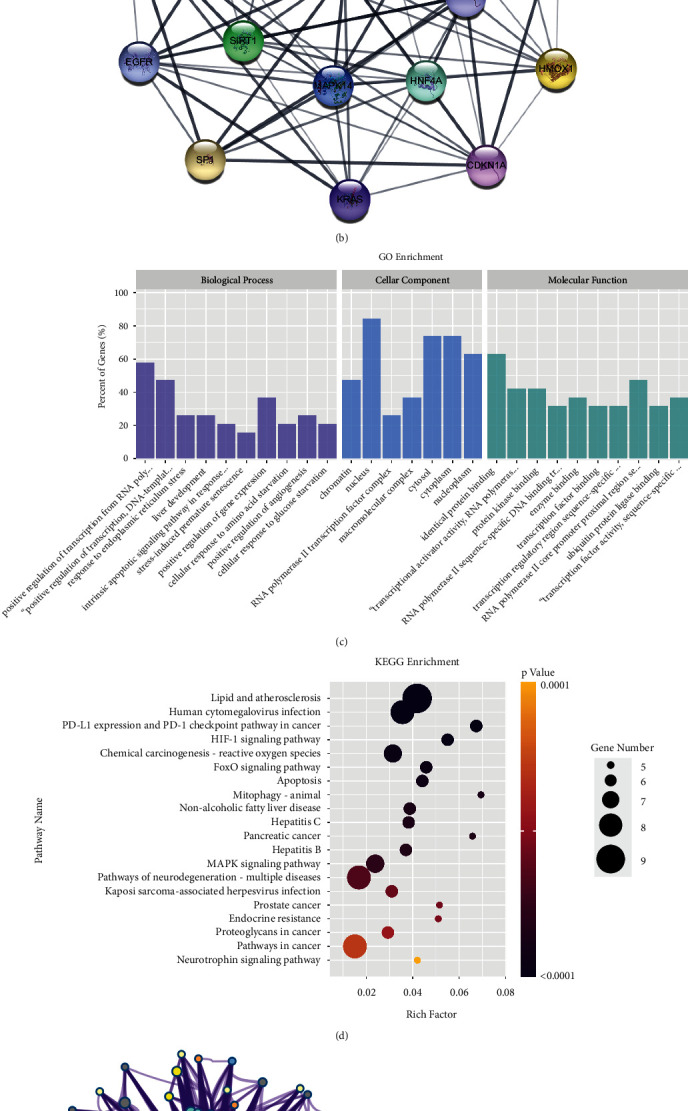
Core-DEGs screening, function, and pathway enrichment analysis. (a) PPI network diagram of 68 DEGs; (b) PPI network diagram of core-DEGs; (c) results of GO functional analysis of core-DEGs; (d) results of KEGG pathway enrichment of core-DEGs; (e) results of BF and pathway enrichment analysis of core-DEGs by Metascape; (f) results of PCA analysis of 19 core-DEGs in the control and stress groups.

The PPI network diagram constructed by STRING analysis is shown in Figure 3(a). 68 DEGs all appear in the PPI network. There are 317 interaction lines between proteins and PPI enrichment *p*-value <1.0 × 10^−16^. After the MCODE function analysis of Cytoscape, a cluster of genes with a high intersection point score (Figure 3(b)) was obtained, with a score of 10.667. There were 96 interaction lines between proteins and 19 central node genes (ASNS, TRIB3, ATF4, EIF2S1, CEBPG, RELA, HSPA5, DDIT3, STAT3, MAP3K5, HIF1A, HNF4A, MAPK14, HMOX1, CDKN1A, KRAS, SP1, SIRT1, EGFR), all of which were up-regulated genes, and they were defined as core-DEGs. Uploaded to the DAVID, 19 core-DEGs have been successfully applied to the GO function and KEGG pathway enrichment analysis. BP mainly involves positive regulation of transcription from RNA polymerase II promoter, positive regulation of transcription, DNA-templated, response to ER stress, liver development, and intrinsic apoptotic signaling pathway in response to ER stress, etc. MF mainly focuses on identical protein binding, transcriptional activator activity, RNA polymerase II transcription regulatory region sequence-specific binding, protein kinase binding, RNA polymerase II sequence-specific DNA binding transcription factor binding, and enzyme binding, etc. CC is statistically significant in chromatin, nucleus, RNA polymerase II transcription factor complex, macromolecular complex, cytosol, cytoplasm, and nucleoplasm (Figure 3(c)). The enrichment result of KEGG pathway is shown in Figure 3(d), which mainly involves lipid and atherosclerosis, human cytomegalovirus infection, PD-L1 expression and PD-1 checkpoint pathway in cancer, hypoxia-inducible factor 1 (HIF-1) signaling pathway, and chemical carcinogenesis—ROS, etc. The analysis of BP and pathways of core-DEGs by Metascape show that these 19 core-DEGs are mainly involved in cellular responses to stress, response of eukaryotic translation initiation factor 2 alpha kinase 1 (EIF2AK1)/heme-regulated inhibitor (HRI) to heme deficiency, response to nutrient levels, cellular response to chemical stress, and regulation of DNA-templated transcription in response to stress, etc. (Figure 3(e)). PCA analysis found that based on the expression of 19 core-DEGs, the control group and the stress group can be successfully identified, indicating that they are expected to be diagnostic indicators of stress injury (Figure 3(f)).

### 3.5. Core-DEGs and Immune Infiltrating Cells

Spearman correlation analysis did not find that MAPK14 was significantly related to immune cells. Other core-DEGs were related to immune cells, including B cells naive, T cells follicular helper, and monocells (Table 2). They were mainly positively correlated with B cells naive and T cells follicular helper and negatively correlated with monocells. It can be seen from the types of infiltrating immune cells that STAT3, MAP3K5, HIF1A, and HNF4A were more closely related to immune infiltration.

### 3.6. Gene–miRNA–lncRNA Interactive Network

**Figure 4:**
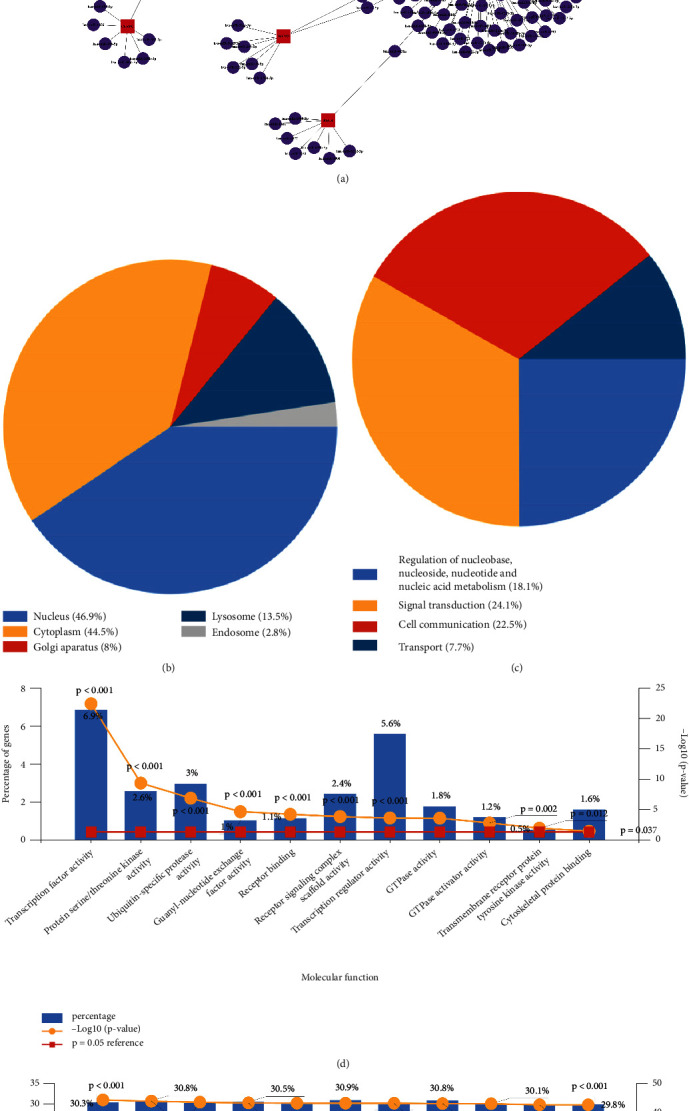
Gene–miRNA–lncRNA interaction network analysis. (a) Results of miRNA prediction of core-DEGs; (b) results of CC enrichment analysis of 283 miRNAs by Funrich; (c) results of BF enrichment analysis of 283 miRNAs by Funrich; (d) results of MF enrichment analysis of 283 miRNAs by Funrich; (e) results of biological pathways enrichment analysis of 283 miRNAs by Funrich.

The miRNA prediction results of 19 core-DEGs are shown in Figure 4(a). Based on our screening criteria, 283 miRNAs were found, including 22 miRNAs with a higher number of cross-linked genes (core-DEGs ≥ 2), as shown in Table 3. Enrichment analysis of 283 miRNAs employed by Funrich showed that CC was significantly enriched in nucleus, cytoplasm, golgi apparatus, lysosome, and endosome (Figure 4(b)); BP focuses on regulation of nucleobase, nucleoside, nucleotide and nucleic acid metabolism, signal transduction, cell communication, and transport (Figure 4(c)); MF mainly involves the transcription factor activity, protein serine/threonine kinase activity, ubiquitin-specific protease activity, guanyl-nucleotide exchange factor activity, receptor binding, receptor signaling complex scaffold activity, transcription regulator activity, GTPase activity, GTPase activator activity, transmembrane receptor protein tyrosine kinase activity, and cytoskeletal protein binding (Figure 4(d)); biological pathways mainly include epidermal growth factor receptor (EGFR/ErbB) signaling network, glypican pathway, sphingosine 1-phosphate (S1P) pathway, tumour necrosis factor-related apoptosis-inducing ligand (TRAIL) signaling pathway, vascular endothelial growth factor (VEGF) and vascular endothelial growth factor receptor (VEGFR) signaling network, beta1 integrin cell surface interactions, platelet-derived growth factor (PDGF) receptor signaling network, proteoglycan syndecan-mediated signaling events, alpha9 beta1 integrin signaling events, interferon (IFN)-gamma pathway, and signaling events mediated by VEGFR1 and VEGFR2, etc. (Figure 4(e)).

Among 22 miRNAs with a high number of cross-linking genes, only miR-27a-3p, miR-155-5p, miR-143-3p, miR-92a-3p, miR-125b-5p, let-7e-5p, miR-145-5p, miR-519d-3p, miR-17-5p, miR-4436a, and miR-24-3p have upstream lncRNAs, which mainly target STAT3, SP1, CDKN1A, and SIRT1. Using StarBase to predict their corresponding lncRNAs, 39 lncRNAs targeting 11 key miRNAs were found, namely NEAT1, MIR22HG, AC005332.7, AC007228.2, XIST, EPB41L4A-AS1, AC084082.1, ERICD, MALAT1, AC087477.2, AC005261.1, NORAD, LINC01772, LINC00240, AC015849.5, MIR155HG, NUP50-AS1, MEG3, JPX, MEOX2-AS1, LZTS1-AS1, LINC00667, AC245884.8, AC245014.3, AC108134.2, AC239868.3, PURPL AC130650.2, AC010980.2, LINC02381, LINC01089, AC027031.2, EBLN3P, DIO3OS, AC124045.1, SNHG4, LINC00265, AP000766.1, and MIRLET7BHG.

### 3.7. Potential Therapeutic Agents for Stress

**Figure 5:**
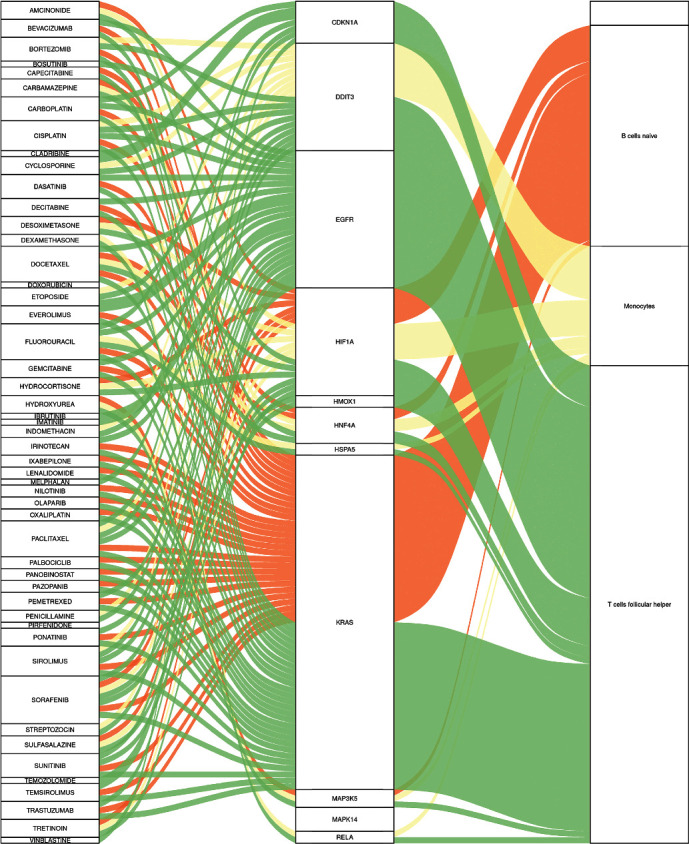
The Sankey diagram between potential therapeutic agents, genes, and immune cells.

85 potential drugs have been identified as targeting 11 core-DEGs, among which CDKN1A, DDIT3, EGFR, HIF1A, HNF4A, and KRAS have relatively rich targeted drugs (Figure 5), which are related to immune cells and are expected to become potential immunotherapeutic targets for stress injury. Docetaxel, fluorouracil, paclitaxel, sirolimus, and sorafenib have been predicted as potential therapeutic drugs for stress, some of which have been proven to have clinical benefits for stress-induced injuries and diseases.

### 3.8. Validation Results of ATF4, EIF2S1, HSPA5, and MAP3K5

**Figure 6:**
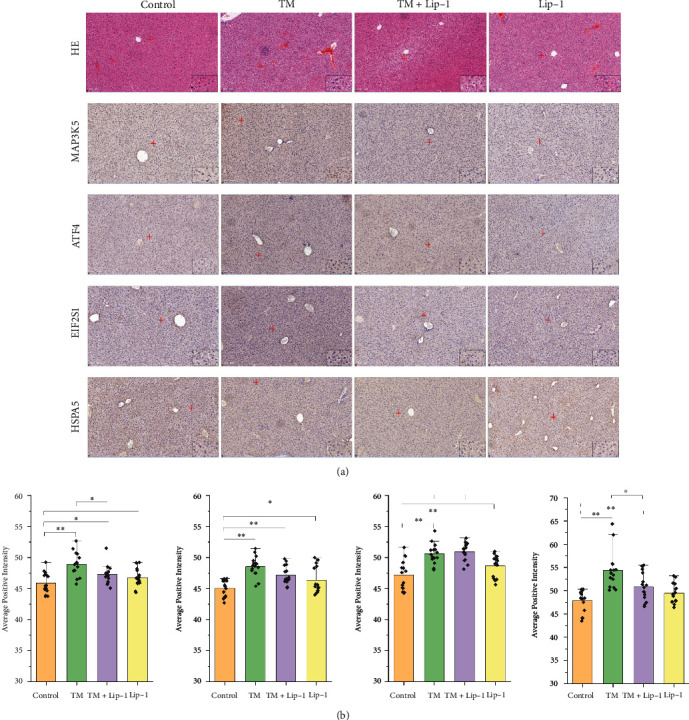
Validation results of ATF4, EIF2S1, HSPA5, and MAP3K5. (a) HE and immunohistochemistry of liver tissue under different groups (magnification, ×200; scale bar, 100 *μ*m; red + marks the position of the magnified image in the lower right corner of the figure); (b) average positive intensity statistics of immunohistochemistry of ATF4, EIF2S1, HSPA5, and MAP3K5 (data presented as the mean ± standard deviation (*n* = 15); LSD in one-way ANOVA was selected for pairwise comparison, ∗∗*P* < 0.01, ∗0.01 < *P* < 0.05, unmarked ∗means *P* > 0.05).

HE staining showed that the hepatic lobule structure was clear in the liver tissue of control mice, and the hepatocyte cords were neatly arranged from the central vein to the surrounding area with less apoptosis, while the hepatic lobule structure was abnormal in mice after intraperitoneal injection of TM, with disorganized hepatocyte cords, cell edema, focal inflammatory cell infiltration in the interstitial space, and a large number of hepatocyte apoptosis. Lip-1 significantly attenuated TM-induced inflammatory cell infiltration, cell cord disorder, and hepatocyte apoptosis without causing significant damage to hepatocytes by itself (Figure 6(a)). Immunohistochemical results showed that TM could lead to increased levels (average positive intensity) of ER stress proteins ATF4, EIF2S1, HSPA5, and MAP3K5. Lip-1, a specific inhibitor of ferroptosis, significantly inhibited the increased levels of ATF4, HSPA5, and MAP3K5 proteins in liver tissues (Figures 6(a) and 6(b)), suggesting that there is a synergistic pathway between ferroptosis and ER stress and that ATF4, HSPA5, and MAP3K5 may be proteins in the common pathway. Although Lip-1 fails to effectively inhibit the increase of EIF2S1 protein, it does not mean that EIF2S1 is not a protein on the common pathway of ferroptosis and ER stress, and the role of EIF2S1 in it needs further investigated.

## 4. Discussion

Since the lab of Dr. Brent R. Stockwell proposed ferroptosis as a form of cell death in 2012 [10], the mechanism of ferroptosis occurrence and its role in disease has become a hot topic of research [19, 20]. Interestingly, pharmacological agents that induce ferroptosis activate the ER stress response, suggesting a crosstalk between ferroptosis and the ER stress response [21, 22]. There is also increasing evidence in recent years that ferroptosis is closely associated with the ER stress response in injury and disease. On the one hand, in ferroptosis induced by ferroptosis inducers, the ER stress is activated simultaneously, and the PERK-eIF2*α* (EIF2S1)-ATF4 pathway inhibited ferroptosis by upregulating HSPA5, system Xc-, and other molecules [22, 23]. On the other hand, the activation of ER stress is involved in the synergistic effect of ferroptosis and apoptosis through the CHOP-p53 upregulated modulator of apoptosis (PUMA) pathway [21, 24]. In addition, in some injuries and diseases, ER stress plays a role in promoting cellular ferroptosis and exacerbating disease damage [25]. Studies have shown that ER stress and ferroptosis are closely related to stress injury [26, 27], but the interaction between ER stress and ferroptosis in stress injury is unknown. Further understanding of the relationship between ferroptosis, ER stress, and apoptosis regulation mechanism is important for clarifying the mechanism of stress injury and screening specific diagnostic indicators.

Our study screened 68 common DEGs for ferroptosis and ER stress using bioinformatics techniques, accounting for approximately 26.255% (68/259) of all ferroptosis genes, suggesting that the ferroptosis pathway may play an important role during ER stress. Through PPI network analysis, we screened 19 up-regulated core-DEGs, and their BP and pathways mainly involved in response to ER stress, lipid and atherosclerosis, cellular response to stress, cellular response to chemical stress, and regulation of DNA-templated transcription in response to stress, etc. Immune cell infiltration analysis revealed a predominance of monocytes, NK cells activated, macrophages M1, T cells CD4 memory resting, and macrophages M0, which is consistent with the pathological changes observed microscopically with focal inflammatory cell infiltration visible between the interstitial spaces of liver tissue. Lip-1 injection significantly attenuated TM-induced inflammatory cell infiltration, cell cord disorder, and hepatocyte apoptosis, but the TM + Lip-1 group still had altered damage and did not fully return to normal compared to the control group, suggesting that there is a partially synergistic pathway between ferroptosis and ER stress, not all of it. From the core-DEGs screened, the ER stress response, especially the PERK–eIF2*α*–ATF4–CHOP pathway, was closely related to ferroptosis. Xu et al. observed iron deposition, lipid radical accumulation, and mitochondrial shrinkage in a mouse model of ulcerative colitis (UC), and also found that the PERK–ATF4–CHOP pathway was significantly activated in the colonic epithelium of UC mice, and that treatment with GSK414, an inhibitor of the ER stress PERK pathway, significantly inhibited the process of ferroptosis [25]. In addition, it has been shown that cigarette smoke condensates (CSC) can activate the ER stress PERK, IRE1*α* pathway, and ferroptosis pathway in human bronchial epithelial cells, and sequencing results showed that the activation of ER stress promoted the occurrence of ferroptosis [28]. It is worth noting that ATF4 seems to be the key link of ER stress affecting ferroptosis, and knockdown of ATF4 in mouse fibroblasts would cause oxidative and iron-dependent cell death [29]. Under the regulation of ATF4, the expression of HSPA5 or system Xc-increased, which enhanced the antioxidant function of GSH/GPX4 [23]. It suggests that the basic level of ATF4 may affect the sensitivity of cells to ferroptosis, and ATF4 may be another important regulator of the ferroptosis pathway. Our validation experiments also showed that Lip-1 significantly inhibited the increase in protein levels of ER stress proteins ATF4, HSPA5, and MAP3K5, suggesting that ATF4, HSPA5, and MAP3K5 may be tandem proteins in the ferroptosis and ER stress pathways, but Lip-1 failed to effectively inhibit the increase of EIF2S1 protein, which is different from the study by Xu and Park et al. [25, 28].

In 19 core-DEGs, in addition to the above crosstalk molecules common to ER stress and ferroptosis, deletion of Bp65 (RELA) was found to significantly up-regulate ferroptosis and exacerbate colitis in IEC-specific NF-kappa Bp65 deficient mice, and phosphorylated Bp65 significantly inhibited ER stress by binding eIF2*α* [25]. In addition to the significant increase in PERK, IRE1*α* pathway, and ferroptosis pathway-related proteins in CSC treated BEAS-2B cells (a normal human bronchial epithelial cell line), the levels of MAP kinase activation (p-ERK, p-p38 (MAPK14), and p-JNK)-related proteins were also significantly enhanced [28]. Juglone can induce the accumulation of Fe^2+^, lipid peroxidation, GSH depletion, up-regulation of HMOX1, and heme degradation to Fe^2+^ in endometrial carcinoma cells, and also participates in inducing autophagy and inhibiting cell migration and ER stress, which may be a new marker for cancer treatment [30]. Tagitinin C induces ER stress and oxidative stress in HCT116 cells, activates Nrf2, and leads to a significant increase in HMOX1 expression, and up-regulated HMOX1 leads to an increase in the pool of unstable iron and promotes lipid peroxidation. Briefly, Tagitinin C induces ferroptosis by activating the PERK–Nrf2–HMOX1 signaling pathway through ER stress [31]. Artesunate (ART) induces ER stress, apoptosis, and ferroptosis via phosphorylation of eIF2*α* in a BON-1 cell line whose sensitivity to ART treatment is associated with long-term differential regulation of p21 (CDKN1A) [32]. Alim et al. found that pharmacological Se enhanced GPX4 and other genes in the selenium group by synergistically activating the transcription factors TFAP2c and SP1, thereby protecting neurons from mitigating the stimulation of ferroptosis and that supplementation with pharmacological selenium effectively inhibited GPX4-dependent ferroptotic, as well as cell death-induced toxicity or ER stress, which are GPX4-independent [33]. However, there are few reports about the network interactions between ASNS, TRIB3, CEBPG, STAT3, MAP3K5, HIF1A, HNF4A, KRAS, SIRT1, EGFR, and other core-DEGs between ER stress and ferroptosis, and further studies are needed.

In summary, the study of molecular network interactions between ER stress and ferroptosis has received increasing attention from researchers in various fields, but stress injury can be caused by a variety of risk factors, with complex symptoms, complex psychological and physiological basis, and is prone to the combination of other injuries and diseases, making it difficult to study the interactions between the two. We try to screen biomarkers and molecular targets related to stress injury by multi-omics (genomics, proteomics, transcriptomics, metabolomics, etc.) and bioinformatics techniques, which are helpful to study the crosstalk between ER stress and ferroptosis in stress injury, and can form a molecular network related to ER stress and ferroptosis, which is more helpful to elucidate the mechanism of stress injury and establish a personalized stress diagnosis system.

## 5. Conclusion

Here, we used bioinformatics technology to screen the intersecting DEGs of ER stress and ferroptosis under stress conditions and analyzed the occurrence, development process, and molecular mechanism of stress injury involved in DEGs, focusing on the linkage between ER stress and ferroptosis. It makes up for the lack of research on the molecular network mechanism of ER stress and ferroptosis in stress injury, and explores the mechanism of stress—ER stress—ferroptosis and molecular network interactions from a novel perspective, which provides a new theoretical basis for the study of stress injury.

## Figures and Tables

**Table 1:** Common DEGs of GSE29929 and ferroptosis.

DEGs	Gene name
Up-regulated (63)	ATF3, PSAT1, CDKN1A, DDIT3, TRIB3, GDF15, JUN, DDIT4, ASNS, CHAC1, ATF4, MYB, SESN2, NNMT, ACSL4, VLDLR, SLC1A4, ARNTL, ATG13, HILPDA, HMOX1, CEBPG, ACSL3, WIPI1, LPIN1, PHKG2, HSPA5, SRXN1, GOT1, EGFR, ATG4D, GABPB1, RELA, SLC7A5, KRAS, SQSTM1, MAPK14, GPT2, SIRT1, SP1, PLIN4, BRD4, EIF2S1, BACH1, WIPI2, PCK2, HERPUD1, MTDH, CS, SLC3A2, SLC2A1, HNF4A, ALOX12B, ELAVL1, ZFP36, MAP3K5, DUSP1, HSF1, HIF1A, STAT3, SETD1B, LONP1, XBP1
Down-regulated (5)	TSC22D3, KLHL24, HSD17B11, SOCS1, ARRDC3

**Table 2:** Spearman correlation analysis between core-EGs and immune cells (R value).

Immune cell	ASNS	TRIB3	ATF4	EIF2S1	CEBPG	RELA	HSPA5	DDIT3	STAT3	MAP3K5	HIF1A	HNF4A	MAPK14	HMOX1	CDKN1A	KRAS	SP1	SIRT1	EGFR
B cells naive	0.750∗	0.500	0.786∗	0.571	0.286	0.643	0.571	0.464	0.714∗	0.714∗	0.786∗	0.821∗	0.517	0.393	0.643	0.857∗	0.464	0.571	0.429
B cells memory	−0.612	−0.204	−0.408	−0.408	−0.204	−0.408	−0.408	−0.612	−0.612	−0.612	−0.408	−0.408	−0.612	−0.204	−0.408	−0.612	−0.612	−0.408	−0.612
Plasma cells	0.464	−0.286	0.143	−0.071	−0.357	0.036	−0.071	−0.036	0.143	0.143	0.143	0.286	0.143	−0.250	0.107	0.286	−0.036	−0.071	0.429
T cells CD8	−0.037	0.482	0.371	0.259	0.296	0.296	0.259	−0.074	0.111	0.111	0.371	0.334	−0.111	0.371	0.222	0.148	−0.074	0.259	−0.296
T cells CD4 naive	−0.204	−0.612	−0.204	−0.204	−0.408	−0.204	−0.204	−0.204	−0.204	−0.204	−0.204	−0.204	−0.408	−0.612	−0.612	−0.408	−0.204	−0.204	0.000
T cells CD4 memory resting	−0.321	−0.214	−0.321	−0.357	−0.500	−0.214	−0.357	−0.286	−0.071	−0.071	−0.321	−0.357	−0.536	−0.143	−0.143	−0.143	−0.286	−0.357	0.000
T cells follicular helper	0.750	0.750	0.750	0.821∗	0.786∗	0.857∗	0.821∗	0.893∗∗	0.893∗∗	0.893∗∗	0.750∗	0.714∗	0.643	0.821∗	0.714∗	0.714∗	0.893∗∗	0.821	0.893∗∗
T cells gamma delta	0.000	0.408	0.408	0.612	0.6112	0.408	0.612	0.612	0.408	0.408	0.408	0.204	0.408	0.204	0.000	0.204	0.612	0.612	−0.204
NK cells resting	0.018	−0.450	−0.468	0.450	−0.468	−0.396	−0.450	−0.144	−0.198	−0.198	−0.468	−0.378	−0.018	−0.288	−0.072	−0.108	−0.144	−0.450	0.288
NK cells activated	0.000	0.464	0.286	0.286	0.464	0.214	0.286	0.071	0.036	0.036	0.286	0.250	0.179	0.357	0.214	0.107	0.071	0.286	−0.321
Monocytes	−0.536	−0.714∗	−0.821∗	−0.929∗∗	−0.786∗	−0.893∗∗	−0.929∗∗	−0.929∗∗	−0.929∗∗	−0.929∗∗	−0.821∗	−0.679∗	−0.536	−0.643	−0.464	−0.643	−0.929∗∗	−0.929∗∗	−0.571
Macrophages M0	0.321	0.214	0.000	0.000	0.000	0.071	0.000	0.250	0.286	0.286	0.000	0.036	0.357	0.321	0.500	0.429	0.250	0.000	0.429
Macrophages M1	0.143	0.143	0.321	0.393	0.464	0.214	0.393	0.286	0.071	0.071	0.321	0.286	0.393	0.000	−0.071	0.036	0.286	0.393	−0.179
Macrophages M2	−0.612	−0.204	−0.408	−0.408	−0.204	−0.408	−0.408	−0.612	−0.612	−0.612	−0.408	−0.408	−0.612	−0.204	−0.408	−0.612	−0.612	−0.408	−0.612
Dendritic cells resting	−0.214	0.286	0.357	0.357	0.286	0.286	0.357	0.071	0.143	0.143	0.357	0.214	−0.143	0.071	−0.143	0.000	0.071	0.357	−0.464
Mast cells resting	−0.429	0.000	0.071	0.321	0.321	0.179	0.321	0.250	0.071	0.071	0.071	−0.143	−0.286	−0.107	−0.536	−0.393	0.250	0.321	−0.250
Neutrophils	−0.204	−0.612	−0.204	−0.204	−0.408	−0.204	−0.204	−0.204	−0.204	−0.204	−0.204	−0.204	−0.408	−0.612	−0.612	−0.408	−0.204	−0.204	0.000

Note: ∗: *p* < 0.1; ∗∗: *p* < 0.01.

**Table 3:** The miRNAs with higher amounts of cross-linked DEGs (*n* ≥ 2).

miRNA	Genes targeted by miRNA	Gene count
miR-892b	SP1, KRAS	2
miR-27a-3p	SP1, KRAS	2
miR-155-5p	SP1, EGFR, SIRT1	3
miR-143-3p	KRAS, HNF4A	2
miR-6875-3p	HNF4A, STAT3	2
miR-92a-3p	STAT3, SIRT1	2
miR-125b-5p	STAT3, CEBPG	2
let-7e-5p	STAT3, CDKN1A	2
miR-4516	STAT3, CDKN1A, TRIB3	3
miR-4650-3p	CDKN1A, HSPA5	2
miR-6756-3p	HSPA5, SP1	2
miR-5196-5p	SP1, CDKN1A	2
miR-145-5p	SP1, CDKN1A	2
miR-202-3p	SP1, CDKN1A	2
miR-6847-5p	TRIB3, CDKN1A	2
miR-519d-3p	EIF2A1, CDKN1A	2
miR-17-5p	EIF2A1, CDKN1A	2
miR-4436a	CDKN1A, RELA	2
miR-5008-5p	CDKN1A, HMOX1	2
miR-6792-3p	SP1, HMOX1	2
miR-24-3p	TRIB3, HMOX1, MAPK14	3
miR-4685-3p	SP1, MAPK14	2

## Data Availability

GSE29929 and ferroptosis genes from NCBI-GEO database and FerrDb database, respectively; additional data are available on request from the corresponding author.

## References

[1] Reser J. E. (2016). Chronic stress, cortical plasticity and neuroecology. *Behavioural Processes*.

[2] Sun S., Zhou J. (2018). Molecular mechanisms underlying stress response and adaptation. *Thoracic Cancer*.

[3] Yi S., Chen K., Zhang L. (2019). Endoplasmic reticulum stress is involved in stress-induced hypothalamic neuronal injury in rats via the PERK–ATF4–CHOP and IRE1–ASK1–JNK pathways. *Frontiers in Cellular Neuroscience*.

[4] Niu S., Shi W., Li Y. (2021). Endoplasmic reticulum stress is associated with the mesencephalic dopaminergic neuron injury in stressed rats. *Analytical Cellular Pathology*.

[5] Marin M. F., Lord C., Andrews J. (2011). Chronic stress, cognitive functioning and mental health. *Neurobiology of Learning and Memory*.

[6] Qie X., Wen D., Guo H. (2017). Endoplasmic reticulum stress mediates methamphetamine-induced blood–brain barrier damage. *Frontiers in Pharmacology*.

[7] Chen X., Cubillos-Ruiz J. R. (2021). Endoplasmic reticulum stress signals in the tumour and its microenvironment. *Nature Reviews. Cancer*.

[8] Qi Z., Chen L. (2019). Endoplasmic reticulum stress and autophagy. *Advances in Experimental Medicine and Biology*.

[9] Ghemrawi R., Khair M. (2020). Endoplasmic reticulum stress and unfolded protein response in neurodegenerative diseases. *International Journal of Molecular Sciences*.

[10] Dixon S. J., Lemberg K. M., Lamprecht M. R. (2012). Ferroptosis: an iron-dependent form of nonapoptotic cell death. *Cell*.

[11] Stockwell B. R., Friedmann A. J., Bayir H. (2017). Ferroptosis: a regulated cell death nexus linking metabolism, redox biology, and disease. *Cell*.

[12] Li J., Cao F., Yin H. L. (2020). Ferroptosis: past, present and future. *Cell Death & Disease*.

[13] Su J., Bian C., Zheng Z. (2022). Cooperation effects of radiation and ferroptosis on tumor suppression and radiation injury. *Frontiers in Cell and Development Biology*.

[14] Lee H., Zandkarimi F., Zhang Y. (2020). Energy-stress-mediated AMPK activation inhibits ferroptosis. *Nature Cell Biology*.

[15] Huang J., Xie H., Yang Y. (2022). The role of ferroptosis and endoplasmic reticulum stress in intermittent hypoxia-induced myocardial injury. *Sleep & Breathing*.

[16] Li W., Li W., Leng Y., Xiong Y., Xia Z. (2020). Ferroptosis is involved in diabetes myocardial ischemia/reperfusion injury through endoplasmic reticulum stress. *DNA and Cell Biology*.

[17] Kuchina A., Brettner L. M., Paleologu L. (2021). Microbial single-cell RNA sequencing by split-pool barcoding. *Science*.

[18] Dewey F. E., Murray M. F., Overton J. D. (2016). Distribution and clinical impact of functional variants in 50,726 whole-exome sequences from the DiscovEHR study. *Science*.

[19] Aguilera A., Berdun F., Bartoli C. (2022). C-ferroptosis is an iron-dependent form of regulated cell death in cyanobacteria. *The Journal of Cell Biology*.

[20] Li S., Huang Y. (2022). Ferroptosis: an iron-dependent cell death form linking metabolism, diseases, immune cell and targeted therapy. *Clinical & Translational Oncology*.

[21] Hong S. H., Lee D. H., Lee Y. S. (2017). Molecular crosstalk between ferroptosis and apoptosis: emerging role of ER stress-induced p53-independent PUMA expression. *Oncotarget*.

[22] Dixon S. J., Patel D. N., Welsch M. (2014). Pharmacological inhibition of cystine-glutamate exchange induces endoplasmic reticulum stress and ferroptosis. *eLife*.

[23] Chen D., Fan Z., Rauh M., Buchfelder M., Eyupoglu I. Y., Savaskan N. (2017). ATF4 promotes angiogenesis and neuronal cell death and confers ferroptosis in a xCT-dependent manner. *Oncogene*.

[24] Ganten T. M., Haas T. L., Sykora J. (2004). Enhanced caspase-8 recruitment to and activation at the DISC is critical for sensitisation of human hepatocellular carcinoma cells to TRAIL-induced apoptosis by chemotherapeutic drugs. *Cell Death and Differentiation*.

[25] Xu M., Tao J., Yang Y. (2020). Ferroptosis involves in intestinal epithelial cell death in ulcerative colitis. *Cell Death & Disease*.

[26] Zhu X., Dong J., Xia Z., Zhang A., Chao J., Yao H. (2017). Repeated restraint stress increases seizure susceptibility by activation of hippocampal endoplasmic reticulum stress. *Neurochemistry International*.

[27] Zhang H., Jiao W., Cui H., Sun Q., Fan H. (2021). Combined exposure of alumina nanoparticles and chronic stress exacerbates hippocampal neuronal ferroptosis via activating IFN-*γ*/ASK1/JNK signaling pathway in rats. *Journal of Hazardous Materials*.

[28] Park E. J., Park Y. J., Lee S. J., Lee K., Yoon C. (2019). Whole cigarette smoke condensates induce ferroptosis in human bronchial epithelial cells. *Toxicology Letters*.

[29] Harding H. P., Zhang Y., Zeng H. (2003). An integrated stress response regulates amino acid metabolism and resistance to oxidative stress. *Molecular Cell*.

[30] Zhang Y. Y., Ni Z. J., Elam E. (2021). Juglone, a novel activator of ferroptosis, induces cell death in endometrial carcinoma Ishikawa cells. *Food & Function*.

[31] Wei R., Zhao Y., Wang J. (2021). Tagitinin C induces ferroptosis through PERK–Nrf2–HO-1 signaling pathway in colorectal cancer cells. *International Journal of Biological Sciences*.

[32] Yan G., Dawood M., Bockers M. (2020). Multiple modes of cell death in neuroendocrine tumors induced by artesunate. *Phytomedicine*.

[33] Alim I., Caulfield J. T., Chen Y. (2019). Selenium drives a transcriptional adaptive program to block ferroptosis and treat stroke. *Cell*.

